# THE IMPLEMENTATION AND RESULTS OF AN INNOVATIVE VIRTUAL EXERCISE PROGRAMS FOR OLDER ADULTS

**DOI:** 10.1093/geroni/igae098.1032

**Published:** 2024-12-31

**Authors:** Eric Levitan, Elizabeth Kemp, Kathryn Porter Starr

**Affiliations:** Vivo, Atlanta, Georgia, United States; Vivo, Atlanta, Georgia, United States; Duke University School of Medicine, Hillsborough, North Carolina, United States

## Abstract

Aging increases the risk of developing sarcopenia, the loss of muscle mass, strength, and function. Sarcopenia, in turn, increases one’s susceptibility to chronic illnesses, including heart disease, cancer, diabetes, arthritis, and dementia, the leading causes of morbidity, mortality, and escalating healthcare expenses in the US. Strength training is a highly effective exercise intervention that reverses age-related muscle loss in older adults2 and restores overall strength and functional abilities. Vivo is an evidence-based program designed using the principle of science of exercise and aging, integrating stretching, balance, cognitive and resistance exercises in a safe, engaging way for older adults. Vivo addresses barriers to strength training through its easy-to-use virtual platform, knowledgeable trainers, engaging social experience and individualized programming that is tailored to meet the participant’s goals and capabilities. Over the past year, Vivo has partnered with several academic centers and community-based organizations to implement virtual exercise programs for older adults. Results from these partnerships show functional improvements and high program satisfaction. This presentation will discuss the steps taken to partner with academic centers and community-based organizations and share quantitative and qualitative outcomes from those partnerships. These results support the results seen in the NIH feasibility study and more importantly, reinforce the growing body of evidence that strength training is an effective strategy to regain muscle strength and improve functional abilities.

